# Extraction of semantic biomedical relations from text using conditional random fields

**DOI:** 10.1186/1471-2105-9-207

**Published:** 2008-04-23

**Authors:** Markus Bundschus, Mathaeus Dejori, Martin Stetter, Volker Tresp, Hans-Peter Kriegel

**Affiliations:** 1 Institute for Computer Science, Ludwig-Maximilians-University Munich, Oettingenstr. 67, 80538 Munich, Germany; 2 Siemens AG, Corporate Technology, Information and Communications, Otto-Hahn-Ring 6, 81739 Munich, Germany; 3 Integrated Data Systems Department, Siemens Corporate Research, 755 College Road East, Princeton, New Jersey 08540, USA

## Abstract

**Background:**

The increasing amount of published literature in biomedicine represents an immense source of knowledge, which can only efficiently be accessed by a new generation of automated information extraction tools. Named entity recognition of well-defined objects, such as genes or proteins, has achieved a sufficient level of maturity such that it can form the basis for the next step: the extraction of relations that exist between the recognized entities. Whereas most early work focused on the mere detection of relations, the classification of the type of relation is also of great importance and this is the focus of this work. In this paper we describe an approach that extracts both the existence of a relation and its type. Our work is based on Conditional Random Fields, which have been applied with much success to the task of named entity recognition.

**Results:**

We benchmark our approach on two different tasks. The first task is the identification of semantic relations between diseases and treatments. The available data set consists of manually annotated PubMed abstracts. The second task is the identification of relations between genes and diseases from a set of concise phrases, so-called GeneRIF (Gene Reference Into Function) phrases. In our experimental setting, we do not assume that the entities are given, as is often the case in previous relation extraction work. Rather the extraction of the entities is solved as a subproblem. Compared with other state-of-the-art approaches, we achieve very competitive results on both data sets. To demonstrate the scalability of our solution, we apply our approach to the complete human GeneRIF database. The resulting gene-disease network contains 34758 semantic associations between 4939 genes and 1745 diseases. The gene-disease network is publicly available as a machine-readable RDF graph.

**Conclusion:**

We extend the framework of Conditional Random Fields towards the annotation of semantic relations from text and apply it to the biomedical domain. Our approach is based on a rich set of textual features and achieves a performance that is competitive to leading approaches. The model is quite general and can be extended to handle arbitrary biological entities and relation types. The resulting gene-disease network shows that the GeneRIF database provides a rich knowledge source for text mining. Current work is focused on improving the accuracy of detection of entities as well as entity boundaries, which will also greatly improve the relation extraction performance.

## Background

**Figure 1:**
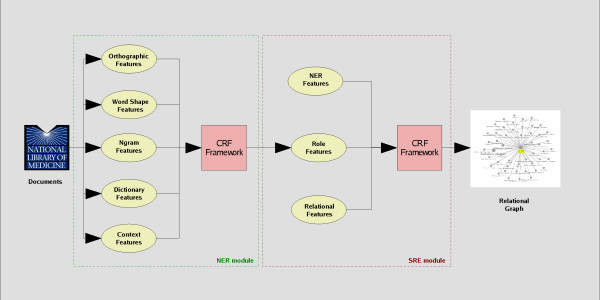
**Cascaded CRF workflow for the combined task of NER and SRE**. In the first module, a NER tagger is trained with the above shown features. The extracted role feature is used to train a SRE model, together with standard NER features and relational features.

The last decade has seen an explosion of biomedical literature. The main reason is the appearance of new biomedical research tools and methods such as high-throughput experiments based on DNA microarrays. It quickly became clear that this overwhelming amount of biomedical literature could only be managed efficiently with the help of automated text information extraction methods. The ultimate goal of information extraction is the automatic transfer of unstructured textual information into a structured form (for a review, see [1]). The first task is the extraction of named entities from text. In this context, entities are typically short phrases representing a specific object such as 'pancreatic neoplasms'. The second logical step is the extraction of associations or relations between recognized entities, a task that has recently found increasing interest in the information extraction (IE) community. The first critical assessments of relation extraction algorithms have already been carried out (see e. g. the BioCreAtIvE II protein-protein interaction benchmark [2] or the TREC 2007 Genomics benchmark [3]). Whereas most early research focused on the mere *detection *of relations, the classification of the *type *of relation is of growing importance [4-6] and the focus of this work. Throughout this paper we use the term 'semantic relation extraction' (SRE) to refer to the combined task of detecting and characterizing a relation between two entities. Our SRE approach is based on the probabilistic framework of Conditional Random Fields (CRFs). CRFs are probabilistic graphical models used for labeling and segmenting sequences and have been extensively applied to named entity recognition (NER). We have developed two variants of CRFs. In both cases, we express SRE as a sequence labeling task. In our first variant, we extend a newly developed type of CRF, the so-called cascaded CRF [7], to apply it to SRE. In this extension, the information extracted in the NER step is used as a feature for the subsequent SRE step. The information flow is shown in Figure 1. Our second variant is applicable to cases where the *key entity *of a phrase is known a priori. Here, a novel one-step CRF is applied that has recently been used to mine relations on Wikipedia articles [8]. The one-step CRF performs NER and SRE in one combined operation.

We compare our two approaches with results obtained by a Support Vector Machine (SVM), a multilayer Neural Network (NN), probabilistic generative models, and with two simplified rule-based methods. We achieve higher or comparable accuracy on two evaluation data sets. In our first experiment, we identify semantic relations between *diseases *and *treatments *from PubMed abstracts using the cascaded CRF model. The detected relations are classified into seven predefined types. In the second experiment, we extract semantic relations between *genes *and *diseases *from GeneRIF [9] sentences (five types of relations) using both the cascaded and the one-step CRF. The cascaded CRF displayed better performance than the one-step CRF. The former was then applied to retrieve gene-disease relationships from the latest human GeneRIF database, validating the scalability of the approach. The extracted network consists of 4939 genes and 1745 diseases connected by 34758 semantic associations and is provided as a resource description framework (RDF) graph. RDF is an important component of the Semantic Web (SW) [10]; thus our work can also be understood as a first step towards shifting unstructured text toward the semantic markup of the biomedical web. The resulting RDF graph serves as an information source for subsequent analyses, for example, finding new gene-disease relationships based on basic graph properties. The work presented by [11] is a promising example of a topology-based analysis, revealing new knowledge implicitly provided by a gene-disease network. Thus, a deeper analysis of our network extracted from textual knowledge in combination with additional biomedical background knowledge might be a promising venue for future research.

### Related Work

Relation Extraction (RE) deals with the problem of finding associations between entities within a text phrase (i. e. usually, but not necessarily, a sentence). Common approaches for relation extraction use rule-based [12], co-occurrence-based [13] and kernel-based [14] methods. In biomedicine, RE has most often been applied to identifying relations between proteins [13,15-18]. [19] focus on detecting associations between proteins and subcellular locations, whereas [20] extract relations between genes, drugs and cell-lines in the context of cancer. Approaches for extracting relations between genes and diseases are less prominent [4,21], however this area is attracting increasing attention.

The different approaches vary in the granularity of the relation extraction process itself. While most studies focus only on detecting relations, a small number of approaches also attempt to extract and characterize the type of relation between entities [4,6,18]. For example, [22] set up an interactive system where NLP methods are applied to generate a set of candidate relationship features, which are evaluated by biological experts to generate a final set of relationship features. [4] set up a system called *SemGen*, which attempts to characterize the semantics of the relations based on whether a gene causes, predisposes, or is simply associated with a disease. In this system, gene entities are identified using existing NER taggers [20,23]. Disease entities are identified with the help of MetaMap [24], a program that maps biomedical text to concepts in the UMLS Metathesaurus [25]. In a subsequent step, each gene-disease pair is classified into one of the relational categories with the help of manually inspected indicator rules. On a test corpus of 1000 sentences a precision of 76% is reported. [26] propose a heuristic post-processing strategy for *SemGen *that aims at selecting the semantic relations that are most likely to be correct. Recently, [5] proposed a method to retrieve genes related to prostate cancer by identifying six gene-prostate cancer relations.

### Data Sets

#### Disease-treatment relation extraction from PubMed abstracts

This annotated text corpus provided by [6] was generated from MEDLINE 2001 abstracts. In a total of 3570 sentences, entities describing diseases and treatments were extracted and disease-treatment relations were classified as *cure, only disease, only treatment, prevents, side effect, vague, does not cure*. Note that, in contrast to the original work, we present results for the full data set, including sentences that contain no entities at all. We believe that this setting is much more realistic than looking only at sentences where at least one of the two entities occurs. The data, enriched with supplementary annotations, are provided online [27].

#### Gene-disease relation extraction from GeneRIF phrases

GeneRIFs [9] are phrases which refer to a particular gene in the Entrez Gene database [28] and describe its function in a concise phrase. Our data set consists of 5720 GeneRIF sentences retrieved from 453 randomly selected Entrez Gene database entries (see Additional file 1 with a list of all Entrez Genes used). The task is to extract and characterize relations between genes and diseases in those sentences. Note that the gene entities themselves are known from the Entrez Gene ID and do not need to be extracted (see section **Methods**). We consider relations describing a wide variety of molecular conditions, ranging from genetic to transcriptional and phosphorylation events:

• **Altered expression: **A sentence states that the altered expression level of a gene/protein is associated with a certain disease or disease state. Example: 'Low expression of BRCA1 was associated with colorectal cancer.'

• **Genetic variation: **A sentence states that a mutational event is reported to be related to a disease. Example: 'Inactivating TP53 mutations were found in 55% of lethal metastatic pancreatic neoplasms.'

• **Regulatory modification: **A sentence associates a disease to a methylation or phosphorylation. Example: 'E-cadherin and p16INK4a are commonly methylated in non-small cell lung cancer.'

• **Any: **A sentence states a relation between a gene/protein and a disease, without any further information regarding the gene's state. Example: 'E-cadherin has a role in preventing peritoneal dissemination in gastric cancer.'

• **Unrelated: **A sentence claims independence between a certain state of a gene/protein and a certain disease. Example: 'Variations in TP53 and BAX alleles are unrelated to the development of pemphigus foliaceus.'

From a biological perspective, methylation and phosphorylation events should be represented as two separate types. However, due to the lack of available examples, we considered both to be of the same type. Two human experts with biological backgrounds annotated the corpus with an inter-annotator agreement estimated of about 84%. A more detailed data set description as well as our annotation guidelines are provided as supplementary data (see Additional file 2). As we did not confine the study to a specific disease model, the labeled disease entities are diverse in terms of the type, ranging from rare syndromes to well studied diseases, primarily cancer and neuro-degenerative diseases like Alzheimer or Parkinson. As mentioned in the **Background**, an entity corresponds usually to a phrase such as 'pancreatic neoplasms'. In our work disease entities were labeled in a way that preserves as much information as possible. For example, tokens specifying the disease like '*lethal metastatic *pancreatic neoplasms', were considered to be part of one disease entity.

## Results and Discussion

### Results

#### Results for disease-treatment relations using PubMed abstracts

In this data set the key entity is not known a priori and the one-step CRF is not applicable. We only report results using the cascaded CRF approach. We benchmark our approach with [6], who compared five different graphical models (GM) and a multilayer neural network for identifying entities and disease-treatment relations. In the first experiment, we compare the CRF for NER with the benchmark methods on the NER task. As in [6], we evaluate two settings for SRE. In the first setting, entities are assumed to be correctly labeled by hand in a preprocessing step and only the existence and the type of the relation between entities needs to be predicted. In the second setting, the entities need to be identified as well. To achieve comparable results we use identical accuracy measures, namely precision, recall and F-measure for NER, and accuracy for SRE. Precision, recall and F-measure are estimated on a token level with the MUC evaluation score [29]. We used 5-fold cross-validation, in accordance with the 80%/20% training/test split used by [6].

Table 1 shows the results for NER and SRE. We achieve an F-measure of 72% on NER identification of disease and treatment entities, wheras the best graphical model achieves an F-measure of 71%. The multilayer NN can not address the NER task, as it is unable to work with the high-dimensional NER feature vectors [6]. Our results on SRE are also very competitive. When the entity labeling is known a priori, our cascaded CRF achieved 96.9% accuracy compared to 96.6% (multilayer NN) and 91.6% (best GM). When the entity labels are assumed to be unknown, our model achieves an accuracy of 79.5% compared to 79.6% (multilayer NN) and 74.9% (best GM).

**Table 1:** Results for the disease-treatment corpus.

	**NER**			**SRE**	
	Recall	Precision	F-score	Accuracy (Entities given)	Accuracy (Entities hidden)
Best GM	-	-	71.0	91.6	74.9
Multilayer NN	-	-	-	96.6	**79.6**
**cascaded CRF**	69.0	75.3	**72.0**	**96.9**	79.5

NER and SRE performance based on evaluation scores proposed by [6]. Relation classification accuracy for seven types of relations is shown for two settings: (1) when the entities are given as gold standard and (2) when the entities have to be extracted. The cascaded CRF outperforms the best GM approach and it shows similar performance to the multilayer NN, where the latter approach can not be applied to the NER task, due to the large feature vectors.

In summary, our cascaded CRF is clearly superior to the best graphical model of [6] in both tasks. The performance on SRE is comparable to the multilayer NN, note however that this method is unable to to be applied to NER.

#### Results for gene-disease relations using GeneRIF sentences

For the second data set a more stringent criterion for evaluating NER and SRE performance is used. As noted earlier, [6] use the MUC evaluation scoring scheme for estimating the NER F-score. The MUC scoring scheme for NER works at the token level, meaning that a label correctly assigned to a specific token is seen as a true positive (TP), except for those tokens that belong to no entity class. SRE performance is measured using accuracy. In contrast to [6], we assess NER as well as SRE performance with an entity level based F-measure evaluation scheme, similar to the scoring scheme of the bio-entity recognition task at BioNLP/NLPBA [30] from 2004. Thus, a TP in our setting is a label sequence for that entity, which exactly matches the label sequence for this entity from the gold standard.

Section **Methods **introduces the terms token, label, token sequence and label sequence. Consider the following sentence: 'BRCA2 is mutated in stage II breast cancer.' According to our labeling guidelines, the human annotators label *stage II breast cancer *as a disease related via a genetic variation. Assume our system would only recognize *breast cancer *as a disease entity, but would categorize the relation to gene 'BRCA2' correctly as *genetic variation*. Consequently, our system would obtain one false negative (FN) for not recognizing the whole label sequence as well as one false positive (FP). In general, this is clearly a very hard matching criterion. In many situations a more lenient criterion of correctness could be appropriate (see [31] for a detailed analysis and discussion about various matching criteria for sequence labeling tasks).

To assess the performance we use a 10-fold cross-validation and report recall, precision and F-measure averaged over all cross-validation splits. Table 2 shows a comparison of three baseline methods with the one-step CRF and the cascaded CRF. The first two methods (*Dictionary+naive rule-based *and *CRF+naive rule-based*) are overly simplistic but can give an impression of the difficulty of the task. Recall, that in this data set NER reduces to the problem of extracting the disease since the gene entity is identical to the Entrez Gene ID. In the first baseline model (*Dictionary+naive rule-based*), the disease labeling is done via a dictionary longest matching approach, where disease labels are assigned according to the longest token sequence which matches an entry in the disease dictionary. The second baseline model (*CRF+naive rule-based*) uses a CRF for disease labeling. The SRE step, referred to as *naive rule-based*, for both baseline models works as follows: After the NER step, a longest matching approach is performed based on the four relation type dictionaries (see **Methods**). Given that exactly one dictionary match was found in a GeneRIF sentence, each identified disease entity in a GeneRIF sentence is assigned with the relation type of the corresponding dictionary. When several matches from different relation dictionaries are found, the disease entity is assigned the relation type which is closest to the entity. When no match can be found, entities are assigned the relation type *any*. The third benchmark method is a two-step approach (*CRF+SVM*), where the disease NER step is performed by a CRF tagger and the classification of the relation is done via a multi-class SVM with an RBF kernel. The feature vector for the SVM consists of relational features defined for the CRF in section **Methods **(Dictionary Window Feature, Key Entity Neighborhood Feature, Start of Sentence, Negation Feature etc.) and the stemmed words of the GeneRIF sentences. The *CRF+SVM *approach was greatly improved by feature selection and parameter optimization, as described by [32], using the LIBSVM package [33]. In contrast to the *CRF+SVM *approach, the cascaded CRF and the one-step CRF easily handle the large number of features (75956) without suffering a loss of accuracy.

**Table 2:** Results for the gene-disease corpus.

	**Recall**	**Precision**	**F-score**
Dictionary + naive rule-based	43.31	42.98	43.10
CRF + naive rule-based	67.62	71.88	69.68
**one-step CRF**	73.36	78.66	75.90
**cascaded CRF**	76.61	79.46	78.00
CRF + SVM	76.63	79.48	78.03

NER and SRE performance comparison of one-step and cascaded CRF with three benchmark methods. The cascaded CRF is on par with the *CRF+SVM *model, where the latter one requires an expensive preceding feature selection step.

In the combined NER-SRE measure (Table 2), the one-step CRF is inferior (F-measure difference of 2.13) when compared to the best performing benchmark approach (*CRF+SVM*). This is explained by the inferior performance on the NER task in the one-step CRF. The one-step CRF achieves only a pure NER performance of 84.27%, while in the *CRF+SVM *setting, the CRF achieves 86.97% for NER.

As shown in table 2, the cascaded CRF is on par with the *CRF+SVM *benchmark model. Table 3 lists the relation-specific performance for the cascaded CRF. Recall from the beginning of this section, that we use an entity-based F-measure to evaluate our results on this data set. Clearly, there is a strong correlation between the number of labeled examples in the training data (see Additional file 2) and the performance on the various relations. For *any*, *altered expression *as well as *genetic variation *relations we exceed the 80% F-measure boundary. Only for two types of relations does accuracy fall below this boundary, namely for *unrelated *and *regulatory modification *relations. This moderate performance can be explained by the relatively low number of available training sentences for these two classes.

**Table 3:** Results Semantic Relation Extraction.

	**Recall**	**Precision**	**F-score**
Any	79.46	78.45	78.95
Unrelated	60.26	70.59	65.02
Altered expression	77.96	79.90	78.91
Genetic variation	77.76	82.45	80.04
Regulatory modification	69.17	73.28	71.16

Overall	76.61	79.46	78.00

NER and SRE performance of the cascaded CRF approach for the five different relation types according to recall, precision and F-measure averaged over the 10 cross-validation test runs.

In general, the CRF model allows for the inclusion of a variety of arbitrary, non-independent input features ranging from simple orthographic to more complex relational features. In section **Methods **we give a detailed description of all features used in our system. To estimate the impact of individual features on the overall performance for the combined NER+SRE score, we trained several one-step CRFs on the same data (one specific cross-validation split), but with different feature settings. In particular, we are interested in the impact of the various relational features. Since the relational feature setting between the two applied types of CRFs was similar, we restrict this evaluation to the one-step model here. Table 4 lists the impact of different features for the one-step CRF model in terms of recall, precision and F-measure. The baseline one-step CRF setting uses features typical for NER tasks, such as orthographic, word shape, n-gram and simple context features. Since we are addressing a relation extraction task, the results are poor, as expected (F-measure 38.48 and 39.65 before and after adding dictionary features, respectively). With the advent of longer/special relational features for the relation task, our system gains a large performance increase (F-measure 67.38 after adding the dictionary window feature). The inclusion of the start window feature (F-measure increase of 4.56) and the key entity neighborhood feature (F-measure increase 2.04) both gain an additionally performance increase. The inclusion of the negation window feature moderately improves recall for the *any *relation and improves precision for *altered expression*, *genetic variation *and *regulatory modification*.

**Table 4:** Evaluation of System Components.

Baseline CRF	•	•	•	•	•	•
Dictionaries		•	•	•	•	•
Dictionary Window			•	•	•	•
Start Window				•	•	•
Key Entity Neighborhood					•	•
Negation Window						•
Recall	35.89	38.13	64.30	70.01	71.81	72.16
Precision	41.47	41.30	70.78	74.00	75.87	78.56
F-score	38.48	39.65	67.38	71.94	73.98	75.22

Contribution of different features to the overall performance of the one-step CRF for the 9th cross-validation run. The baseline model includes orthographic, word shape, n-gram and the basic context feature.

#### Results gene-disease network from the complete GeneRIF database

The trained cascaded CRF model was applied to the latest GeneRIF version, consisting of a total of 110881 human GeneRIFs^1^. Gene-disease relations were identified and stored in a relational database in approximately six hours on a standard Linux PC with an Intel Pentium IV processor, 3.2 GHz. To provide the resulting information in a structured manner, we normalized each identified disease name by mapping it to a MeSH ontology entry. We thereby applied a simple reference resolution strategy: First, we tried to map each identified disease to a MeSH entry's name or to one of its synonyms. If the disease did not match an ontology entry, we iteratively decreased the number of tokens until the token sequence matched a MeSH entry. A reference resolution for gene names is not needed since the GeneRIF ID is known (see **Methods **for details). With this mapping strategy 34758 of the 38568 disease associations could be mapped to an appropriate MeSH entry, resulting in a gene-disease graph with a total of 34758 semantic associations between 4939 unique genes and 1745 unique disease entities.

**Figure 2:**
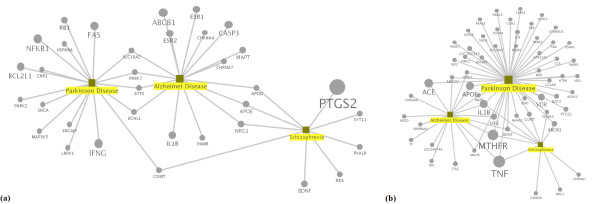
**Sample subgraphs of the gene-disease graph**. Diseases are shown as squares, genes as circles. The entities for which associations are extracted, are highlighted in yellow. We restricted ourselves to genes, which our model inferred to be directly associated with Parkinson's disease, regardless of the relation type. The size of the nodes reflects the number of edges pointing to/from this node. Note that the connectivity is calculated based on the entire subgraph, whereas (a) shows a subgraph restricted to altered expression relations for Parkinson, Alzheimer and Schizophrenia and (b) shows a genetic variation subgraph for the same diseases.

Edges in the graph represent the predefined types of relations defined earlier, while nodes represent diseases or genes, respectively. According to the predefined types of relations, several edges between a gene and a disease can exist. This would be e. g. the case if a publication reports a mutation of a gene in a disease, while another research paper reports high expression levels of that gene in the same disease. Several different filtering steps can be applied to the complete RDF graph, resulting in subgraphs conditioned on e. g. specific diseases, genes or relation types. Assume e. g. that we are interested in the genetic relationship between Parkinson's disease and other diseases (e. g. Alzheimer and Schizophrenia, see Figure 2). In the first filter step, we only consider genes that our model identified to be associated with Parkinson's disease. Our model extracted 97 genes in total for the five types of relations. With these 97 genes, 601 other diseases were linked. Subsequently, all genes were included that were associated with those diseases. Note, that we are only interested in the relationships between Parkinson's disease, Alzheimer and Schizophrenia. Therefore, we exclude all other disease entities and the genes linked with them. Finally, subgraphs are created for the relation type 'altered expression' Figure 2(a) and 'genetic variation' Figure 2(b). The size of the nodes represents the degree of a node (i. e. the number of links the node has to other nodes with respect to the selected relation). As can be seen from Figure 2, the degree of nodes may vary significantly across different views. For example, gene PTGS2 shows a much higher degree in the 'altered expression' graph than in the 'genetic variation' graph. A gene node with high degree shows an association with a multitude of different diseases present in the graph under consideration. This indicates that such a gene is a strong subject of discussion in the literature, in contrast to sparsely connected genes in the graph, constructed for a set of certain types of relations and a certain set of diseases. If such a literature-derived gene-disease network follows a scale-free distribution, as it was shown for the human gene-disease network [34] based on experimentally validated relationships from OMIM™ database, new links could be more likely between these highly-discussed hubs and disease entities. Indeed, in the latest GeneRIF set, not used in our experiments, PTGS2 is mentioned as being associated with Parkinson's disease due to altered expression.

The resulting noisy graph is provided as a resource description framework (RDF) graph [35] (see Additional file 3). Thus, the association network is represented in terms of RDF triplets, i. e. subject (gene), predicate (association) and object (disease) using the Bio2RDF [36] URIs as unique identifiers for genes and diseases.

## Discussion

In this paper we addressed the problem of extracting semantic biomedical relations with a sequence labeling approach, based on conditional random fields. CRFs are known to easily incorporate a rich set of features without negatively affecting prediction accuracy [37] Thus there is no need for expensive preprocessing, such as feature selection. Two variants were developed, the cascaded CRF and the one-step CRF. We benchmarked our approach on two different data sets with different underlying properties. The first data set concentrates on mining relations from general free text, such as PubMed abstracts or full text articles. In this type of text, only the cascaded CRF can be applied. In the cascaded CRF models, the identified entities from the NER step are used as a feature for the subsequent SRE step (Figure 1). This is exactly where the difference between our approach and the classical view of problem lies, whereby the extracted entities are usually fixed after the first step and the only remaining task is to assign the pair to the most likely relation type. The second data set contains concise phrases, created by domain experts. A particular feature of the second data set is that the investigated text phrase refers to a key entity (see **Methods**). In this data set both the cascaded CRF and the one-step CRF can be applied. In the one-step CRF, NER and SRE step are merged together resulting in faster training. Unfortunately the performance was inferior to the cascaded CRF and other benchmark methods.

### Disease-treatment relations from PubMed abstracts

The performance of the cascaded CRF on the data set provided by [6] is on par with the multilayer NN and superior to the best GM. This may be due to the discriminative nature of CRFs and NNs, which could be an advantage over the generative GM. Moreover, it should be stated that the multilayer NN does not scale well with the number of features, limiting its applicability. In [6] the NN could not be applied to the NER task, due to the large feature vectors. Our approach can be applied to both tasks, NER and SRE, achieving very competitive results. In contrast to [6], however, we do not make any use of syntactic higher-level features, such as Part-Of-Speech (POS) tags or Noun Phrase (NP) chunks. When the entities are already given for the SRE task, our approach achieves very accurate results, with an increase in accuracy of 18 percentage points, compared to the case where the entities were hidden and had to be recognized as well. Consequently, the most potential for further improvement lies in the correct identification of treatment and disease entities, since accuracy significantly decreases when the entities need to be identified and were not given a priori. This is especially true for treatment entities, where performance of identifying treatments is only 64.85% (F-measure), compared to disease NER performance of 77.20% (F-measure). Thus, most errors in SRE do occur when e. g. in the NER step a treatment entity was missed, resulting in a consecutive error of the following SRE step. Since the definition of treatments is in general vague, possible improvements could be achieved with the inclusion of a larger and/or more refined treatment dictionary. Currently, all entries of the D MeSH branch are simply used to fill the treatment dictionary, while [6] stress the careful inclusion of subbranches of the MeSH ontology.

### Gene-disease associations from GeneRIF phrases

On the GeneRIF data set the cascaded CRF performs as well as the *CRF+SVM *model. However it should be noted that training of the cascaded CRF is much faster (factor of ten in our setting), since no time-consuming feature selection is needed. The one-step CRF cannot cope with the above mentioned methods, primarily as a result of a lower recall in the NER step. An investigation of different feature weights revealed a stronger dominance of relational features in the one-step CRF compared to the cascaded CRF. Thus, the absence of certain relational features hurts the NER performance of the one-step CRF, because the relational features are a strong indicator of an occurring disease entity in this model. The fact that for *any *relations, where our relational features are usually switched off, the performance decrease is highest (F-measure difference 1.7, compared to the cascaded CRF) supports this hypothesis. For the remaining types of relations, the one-step model can cope with the benchmark approach.

Major improvements for both approaches can be achieved with a more accurate detection of entity boundaries. The overall system performance significantly increases when relaxing the hard matching criterion to softer ones (as presented in [31]). This implies that many entity boundaries are not identified properly. On the one side, this could be partly due to labeling inconsistencies of the human annotators. On the other side, it might originate from the labeling guidelines of diseases. All variable descriptions of a particular disease, such as the form '*non-small *cell lung cancer' or '*stage I-III *endometrial cancer' had to be identified, as well as directly adjacent prepositional phrases like 'cancer *of the lung*'. This makes the task clearly more challenging. The F-measure for a soft matching criterion, when only a part of an entity has to be detected properly, increases to 85.20% (F-measure) (NER+SRE). Another performance increase can be obtained with a more accurate detection of *unrelated *relations. In our framework an *unrelated *relation is a gene-disease pair for which a phrase states that the two entities are not related to each other under a specific setting.

In contrast to previous studies, where *unrelated *relations are most often skipped, we decided to categorize them, since our corpus contains about 7% unrelated statements, which is roughly three times higher than in the work of [4]. However, for a supervised learning approach this is still a very sparse training set, resulting in a low accuracy. The same problem holds for *regulatory modification *relations, where the poor performance is again likely due to the small amount of available examples in our corpus (only 3.5% of the total number of relations). Thus, for both types of relations we expect a significant increase in performance with the inclusion of more training data.

Regarding the definition of the gene-disease relation types, we emphasize that they do not account for the etiological property underlying a specific gene-disease relation. Thus, whether or not a gene is causing the disease or is just associated with the disease pathogenesis is not encoded in the gene-disease relationships defined here. However, our predefined types and the gene-disease relations extracted on that basis can provide helpful information for further biomedical research (e. g. annotation of experiments or providing additional information for experiment design). For the identification of biomarker candidates, the information on which level of the biological dogma (e. g. DNA, RNA, protein etc.) molecules are discriminative for a certain disease, provides highly valuable information, independent of their role in the disease etiology [38]. Nevertheless, we plan to extend our relation types towards etiological information as proposed by [4].

Yet another issue is that we focus on extracting the relations and their types between entities and do not take into account additional information, such as the conditions/properties under which a relation holds. For example, when extracting associations between diseases and genes, it is important to know that certain facts hold for specific populations only. Incorporating these conditions into the relation extraction task, will require deeper syntactic analysis of the sentences. This is an aspect of our ongoing research.

### Gene-disease network

To validate the large-scale applicability of our SRE approach we mined all sentences from the latest human GeneRIF database and retrieved a gene-disease network for five types of relations. As already noted, this network is a noisy representation of the 'true' gene-disease network due to the fact that the underlying source was unstructured text. Nevertheless even though only mining the GeneRIF database, the extracted gene-disease network reveals that a lot of additional knowledge lies buried in the literature, which is not yet reported in databases (the number of disease genes from GeneCards [39] is 3369 as of August 8th, 2007). Removing the genes which only have negative associations labels, results in a set of 4856 genes in our complete graph. Of course, this resulting gene set does not consist exclusively of disease genes. However, a lot of potential knowledge lies in the literature derived network for further biomedical research, e. g. for the identification of new biomarker candidates.

In the future we are planning to replace our simple mapping strategy to MeSH with a more advanced reference resolution approach. If a labeled token sequence could not be mapped to a MeSH entry, e. g. 'stage I breast cancer', then we iteratively decrease the number of tokens, until we obtained a match. In the mentioned example, we would get an ontology entry for breast cancer. Of course, this mapping is not perfect and is one source of errors in our graph. E. g. our model often tagged 'oxidative stress' as disease, which is then mapped to the ontology entry stress. Another example is the token sequence 'mammary tumors'. This phrase is not part of the synonym list of the MeSH entry 'Breast Neoplasms', while 'mammary neoplasms' is. As a consequence, we can only map 'mammary tumors' to 'Neoplasms'.

In general, criticism could be expressed against analyzing GeneRIF sentences rather than making use of the enormous information available from original publications. However, GeneRIF phrases are of high quality, as each phrase is either created or reviewed by MeSH (Medical Subject Headings) indexers, and the number of available sentences is growing rapidly [40]. Thus, analyzing GeneRIFs might be advantageous compared to a full text analysis, as noise and unnecessary text is already filtered out. This hypothesis is underscored by [41], who set up an annotation tool for microarray results based on two literature databases: PubMed and GeneRIF. They conclude that a number of benefits resulted from using GeneRIFs, including a significant decrease of false positives as well as an apparent reduction of search time. Another study highlighting advantages resulting from mining GeneRIFs is the work of [42].

## Conclusion

We propose two new methods for the extraction of biomedical relations from text. We introduce cascaded CRFs for SRE for mining general free text, which has not been previously studied. In addition, we use a one-step CRF for mining GeneRIF sentences. In contrast to previous work on biomedical RE, we define the problem as a CRF-based sequence labeling task. We demonstrate that CRFs are able to infer biomedical relations with fairly competitive accuracy. The CRF can easily incorporate a rich set of features without any need for feature selection, which is one its key advantages. Our approach is quite general in that it may be extended to various other biological entities and relations, provided appropriate annotated corpora and lexicons are available. Our model is scalable to large data sets and tags all human GeneRIFs (110881 as of August 8th 2007) in a fairly moderate amount of time (approximately six hours). The resulting gene-disease network shows that the GeneRIF database provides a rich knowledge source for text mining.

## Methods

Our goal was to develop a method that automatically extracts biomedical relations from text and that classifies the extracted relations into one of a set of predefined types of relations. The work described here treats RE/SRE as a sequential labeling problem typically applied to NER or part-of-speech (POS) tagging. In what follows, we will formally define our approaches and describe the employed features.

### Semantic Relation Extraction as sequence labeling task

Sequential labeling tasks are also known as *sequential supervised learning problems *[43] and can be formulated as follows:

Let (**x**,**y**) denote a pair of sequences where the tokens *x*_1_, *x*_2_, ⋯, *x*_*n *_are words and *y*_1_, *y*_2_, ⋯, *y*_*n *_are token labels or tags. The complete training set consists of *M *such sequence pairs. The goal is to build a classifier *c *that correctly predicts a new label sequence **y **= *c*(**x**) given the input sequence **x**. Whereas in NER *y*_*i *_denotes the entity class of *x*_*i*_, in SRE *y*_*i *_denotes both the entity class and the relation class of *x*_*i*_. Tokens, which are not part of a named entity, are marked as outside (see Additional file 2).

### Conditional Random Fields

Formally a CRF can be defined as an undirected graphical model with vertices *Y*_1_, *Y*_2_, ⋯, *Y*_*n *_representing random variables and edges representing conditional dependencies. Hereby, the random variables are assumed to be conditionally dependent on a set of input variables *X*_1_, *X*_2_, ⋯, *X*_*n*_. For sequence modeling, it is assumed that *Y*_*i *_only has edges to its predecessor *Y*_*i*-1 _and successor *Y*_*i*+1_, thus obtaining a linear Markov chain. The conditional probability of a label or state sequence given an input sequence is defined as

(1) $$P(y|x)=\frac{1}{Z_{x}}\exp\left(\sum_{j=1}^{N}\sum_{k=1}^{K}\lambda_{k}f_{k}(y_{j-1},y_{j},x,j)\right)$$

where *Z*_*x *_is a normalization factor, *f*_*k*_(*y*_*j*-1_, *y*_*j*_, **x**, *j*) is an arbitrary feature function, *K *is the number of feature functions and λ_*k *_is a learned weight for each feature function and can range from -∞ to ∞, and *N *is the length of the input sequence. Each feature function *f*_*k *_represents the strength of interaction between subsequent labels, dependent on the input sequence. The corresponding feature weight λ_*k *_specifies whether the association should be favored or disfavored: Higher values of λ make their corresponding label transitions more likely. The weights are learned from labeled training data by Maximum Likelihood Estimation (MLE). The normalization factor *Z*_*x *_is the sum over all possible state or label sequences *S*^*N*^,

(2) $$Z_{x}=\sum_{s\in S^{N}}\exp\left(\sum_{j=1}^{N}\sum_{k=1}^{K}\lambda_{k}f_{k}(y_{j-1},y_{j},x,j)\right).$$

Labeling a new unseen token sequence is done via a Viterbi algorithm which finds the most likely label sequence according to equation (1). For more details on CRFs, see [44].

#### SRE as a cascaded sequence labeling problem

A typical example for a cascaded approach in natural language processing (NLP) is noun-phrase chunking. Here, the part-of-speech (POS) tags are derived by a trained tagger, in an intermediate step. In a second step the noun-phrases are extracted, where the output of the first model serves as features for the second task.

In the cascaded SRE, two CRFs are trained: a CRF for NER and a second CRF for SRE. The trained CRF for NER is first applied to identify all entities of interest. These entities are then used as additional input features to help solve the SRE problem (Figure 1). Consider the following sentence from the disease-treatment corpus: 'We investigated the hypothesis that an antichlamydial macrolide antibiotic, roxithromycin, can prevent or reduce recurrent major ischaemic events in patients with unstable angina'. In the first step, the treatment entity (antichlamydial macrolide antibiotic, roxithromycin) and the disease (unstable angina) are extracted by a NER CRF (see [6] for labeling guidelines). Thus, in the first step the task is to identify the labels *disease *and *treatment *for the corresponding tokens. The second CRF then identifies the relational labels *disease_prev *and *treatment_prev *based on the features derived for the first CRF and features representing the identified entities. Note, that a relation is represented as labels of the involved entities.

#### SRE as a one-step sequence labeling problem

Here we only consider text phrases that refer to a *key entity*. All other entities in the text phrase, so-called *secondary entities*, are assumed to be related to the key entry. Thus, a secondary entity's label encodes the type of the entity plus the type of relation with the key entity. Note, that NER and SRE are solved jointly in one step. For example, [8] mined biographical texts, where the above stated assumption about a key entity holds. GeneRIF sentences represent a similar style of text in the biomedical domain: They describe the function of a gene/protein, the key entity, as a concise phrase. Consider the following GeneRIF sentence linked to the gene COX-2: 'COX-2 expression is significantly more common in endometrial adenocarcinoma and ovarian serous cystadenocarcinoma, but not in cervical squamous carcinoma, compared with normal tissue.' This sentence states three disease relations with COX-2 (the key entity), namely two altered expression relations (the expression of COX-2 relates to endometrial adenocarcinoma and ovarian serous cystadenocarcinoma) and one unrelated relation (cervical squamous carcinoma).

#### System Description

We use the MALLET [45] package, which provides an efficient implementation for CRFs. We used linear-chain CRFs and used the default Gaussian prior provided by MALLET.

The structure of the CRF in our setting is given by a linear-chain CRF (see e. g. [46] for a graphical representation). Tokens not belonging to any entities are marked as outside, while word tokens belonging to an entity (i. e. diseases, treatments) are labeled with the type of the entity plus the relation type for this entity. In addition, a flag is set whether or not a token marks the beginning of an entity. Certain state transitions are constrained by default, as done in some NER approaches [37,47], e. g. the transition from inside an entity to the beginning of an entity is excluded by definition.

The simplest features are the word tokens themselves (no stemming performed). We do not use any higher level syntactic features like POS tags or NP chunks. Besides the word features, we primarily make use of features which are extracted from the tokens themselves and which are described in the following paragraph. Note that the features are used in both types of CRFs (one-step and cascaded), unless explicitly stated otherwise. Features at the token level, e. g. orthographic, word shape, n-gram, dictionary and simple context features, have been extensively used in the IE community and have become a standard feature set for machine learning based IE approaches (see e. g. [6-8,18,37,47-49]).

#### Orthographic Features

Biomedical entities often yield some orthographic characteristics: They often consist of capitalized letters, include digits or are composed of combinations of both. Thus, these features are helpful in distinguishing various types of biomedical entities. These features can be easily implemented using regular expressions. The set of regular expressions used in this work is displayed in Table 5.

**Table 5:** Orthographic Features.

**Orthographic Feature**	**Regular Expression**
Init Caps	[A-Z].*
Init Caps Alpha	[A-Z][a-z]*
All Caps	[A-Z]+
Caps Mix	[A-Za-z]+
Has Digit	.*[0-9].*
Single Digit	[0-9]
Double Digit	[0-9][0-9]
Natural Number	[0-9]+
Real Number	[-\+][[0-9]+[\.,]+[0-9].,]+
Alpha-Numeric	[A-Za-z0-9]+
Roman	[ivxdlcm]+|[IVXDLCM]+
Has Dash	.*-.*
Init Dash	-.*
End Dash	.*-
Punctuation	[,\.;:\?!-\+"]
Greek	(alpha|beta|...|omega)
Has Greek	.*\b(alpha|beta|...|omega)\b.*
Mutation Pattern	\w*\d+-*\D+

Orthographic features and their corresponding regular expressions used in the experiments.

#### Word Shape Features

Some words belonging to the same entity class might have the same word shape. For instance, it may be common for disease abbreviations, that digits and letters cannot appear together in the token, while for genes and proteins the co-occurrence of digits and letters is striking.

#### NGram Features

We also used character n-gram word features for 2 ≤ *n *≤ 4. These features help to recognize informative substrings like 'ase' or 'homeo', especially for words not seen in training.

#### Dictionary Features

Since we are tackling two tasks of IE, namely NER and SRE, two classes of dictionaries are employed: (1) entity dictionaries consisting of controlled vocabularies and (2) relation dictionaries, which contain indicative keywords for types of relations.

The disease dictionary is based on all names and synonyms of concepts covered by the disease branch (C) of the MeSH ontology. In addition, a treatment dictionary is introduced for the disease-treatment extraction task, composed of all names and synonyms of concepts from the MeSH D branch. We defined four relation dictionaries for the GeneRIF data set, each composed of relation type specific keywords for the following types of relations: *altered expression*, *genetic variation*, *regulatory modification *and *unrelated*. For example, the *genetic variation *dictionary contains words like 'mutation' and 'polymorphism'. For disease-treatment relations we set up dictionaries containing keywords for *prevent *and *side effect *relations. The relation specific dictionaries are provided as supplementary data (see Additional file 4 and 5).

In general, a dictionary feature is active if several tokens match with at least one entry in the corresponding dictionary. Note that the presence of a certain dictionary entry in a sentence is indicative, but not imperative, for a specific entity or relation. This property is elegantly handled by the probabilistic nature of our approach.

#### Context Features

These features take into account the properties of preceding or following tokens for a current token in order to determine its relation. Context features are very important for several reasons. First, consider the case of nested entities: 'Breast cancer 2 protein is expressed ...'. In this text phrase we do not want to identify a disease entity. Thus, when trying to determine the correct label for the token 'Breast' it is very important to know that one of the following word features will be 'protein', indicating that 'Breast' refers to a gene/protein entity and not to a disease. In our work, we set the window size to three for this simple context feature.

The importance of context features not only holds for the case of nested entities but for RE/SRE as well. In this case, other features for preceding or following tokens may be indicative for predicting the type of relation. Thus, we introduce additional features which are very helpful for determining the type of relation between two entities. These features are referred to as relational features throughout this paper.

#### Dictionary Window Feature

For each of the relation type dictionaries we define an active feature, if at least one keyword from the corresponding dictionary matches a word in the window size of 20, i. e. -10 and +10 tokens away from the current token.

#### Key Entity Neighborhood Feature (only used for one-step CRFs)

For each of the relation type dictionaries we defined a feature which is active if at least one keyword matches a word in the window of 8, i. e. -4 and +4 tokens away from one of the key entity tokens. To identify the position of the key entity we queried name, identifier and synonyms of the corresponding Entrez gene against the sentence text by case-insensitive exact string matching.

#### Start Window Feature

For each of the relation type dictionaries we defined a feature which is active if at least one keyword matches a word in the first four tokens of a sentence. With this feature we address the fact that for many sentences important properties of a biomedical relation are mentioned at the beginning of a sentence.

#### Negation Feature

This feature is active, if none of the three above mentioned special context features matched a dictionary keyword. It is very helpful to distinguish *any *relations from more fine-grained relations.

To keep our model sparse the relation type features are based solely on dictionary information. However, we plan to integrate further information originating, for example, from word shape or n-gram features. In addition to the relational features just defined, we set up additional features for our cascaded approach:

#### Role Feature (only used for cascaded CRFs)

This feature indicates, for cascaded CRFs, that the first system extracted a certain entity, such as a disease or treatment entity. This means, that the tokens that are part of an NER entity (according to the NER CRF) are labeled with the type of entity predicted for the token.

#### Feature Conjunction Feature (only used for cascaded CRFs and only used in the disease-treatment extraction task)

It can be very helpful to know that certain conjunctions of features do appear in a text phrase. E. g., to know that several disease and treatment role features do occur as features in conjunction, is important to make relations like *disease only *or *treatment only *for this text phrase quite unlikely.

## List of Abbreviations Used

CRF, Conditional Random Field; GM graphical model; FN, False Negative; FP, False Positive; IE, Information Extraction; MeSH, Medical Subject Heading; MUC, Message Understanding Conference; NER, Named Entity Recognition; NN, Neural Network; POS, Part-Of-Speech; RDF, Resource Description Framework; RE, Relation Extraction; SRE, Semantic Relation Extraction; SVM, Support Vector Machine; SW, Semantic Web; TN, True Negative; TP, True Positive

## Note

^1^downloaded on August 8th 2007

## Supplementary Material

Additional file 1 Entrez Gene identifiers used in the gene-disease data set. This files contains a list of the 453 randomly selected Entrez Gene database entries for the creation of the gene-disease data set. Click here for file

Additional file 2 Gene-disease data set description. This file provides further details about the gene-disease data set, its creation and the labeling procedure including annotation guidelines and inter-annotator agreement. Click here for file

Additional file 3 GeneRIF gene-disease graph. The gene-disease network extracted from the latest GeneRIF version with a total of 34758 semantic associations between 4939 unique genes and 1745 unique disease entities provided as a resource description framework (RDF) graph. Click here for file

Additional file 4 Keywords of the relation specific dictionary for the gene-disease data set. List of keywords used in the relation specific dictionaries for the gene-disease data set. Click here for file

Additional file 5 Keywords of the relation specific dictionary for the disease-treatment data set. List of keywords used in the relation specific dictionaries for the disease-treatment data set. Click here for file
