# Supplementary material for: Extraction of semantic biomedical relations from text using conditional random fields
Source: BMC Bioinformatics. 2008 Apr 23;9:207. doi: 10.1186/1471-2105-9-207 (PMC2386138; doi:10.1186/1471-2105-9-207)
Supplement: Additional file 2 — Gene-disease data set description. This file provides further details about the gene-disease data set, its creation and the labeling procedure including annotation guidelines and inter-annotator agreement. [file 1471-2105-9-207-S2.pdf]

Data set description

Extraction of semantic biomedical relations from text using conditional random fields

Markus Bundschus[1,2], Mathaeus Dejori[2,3], Martin Stetter[2], Volker Tresp[2] and Hans-Peter Kriegel[1]

[1] Institute for Computer Science, Ludwig-Maximilians-University Munich, Oettingenstr. 67, 80538 Munich, Germany

[2] Siemens AG, Corporate Technology, Information and Communications, Otto-Hahn-Ring 6, 81739 Munich, Germany

[3] Integrated Data Systems Department, Siemens Corporate Research, 755 College Road East, Princeton, New Jersey 08540, USA

Email:
Markus Bundschus – bundschu@dbs.ifi.lmu.de
Mathaeus Dejori – mathaeus.dejori@siemens.com
Martin Stetter – stetter@siemens.com
Volker Tresp – volker.tresp@siemens.com
Hans-Peter Kriegel – kriegel@dbs.ifi.lmu.de

Overview

This in-house developed corpus was created to reflect the task of identifying relations holding between genes and diseases from GeneRIF sentences. In what follows, further details about this data set, its creation, the labeling procedure as well as guidelines for the human annotators are given.

General description

The here described corpus consists of 5.720 sentences extracted from 453 Entrez [2] gene entries. The corresponding Entrez gene id's are provided as supplementary data as well. The corpus was generated to evaluate our relationship extraction approach to identify relations holding between genes and diseases and to extract the relation type. We defined the type for a specific gene-disease relation to be based on the information at which level of molecular activity (DNA, mRNA, protein etc.), a gene is mentioned to be related with a specific disease. Thus, we defined the following assertions: a gene/protein can be in a state of *altered expression, genetic variation or regulatory modification.* We are trying to cover all molecular conditions with these types of relations ranging from genetic to transcriptional up to phosphorylation events, a gene/protein is hypothesized to be associated with a specific disease. Moreover, if no specific information about the molecular state is available, a gene/protein can have *any* relation or, if a relation to a disease is negated in a specific sentence, be *unrelated* with a disease. See section **Annotation** for the definition of predefined types of relations.

Table1 lists the total number of semantic relations in the corpus. According to Table 1, a total of 5.396 relations occur in the data set. 4.516 of the 5.720 sentences have at least one disease mention and thus at least one type of relation to the corresponding Entrez gene given by the GeneRIF. The remaining 1.204 sentences have no disease mentions and thus have no relation. Of the 5.396 disease mentions, a total of 2.253 are non-redundant (120 disease abbreviations). However, this number does not reflect the distinct number of disease mentions which can be mapped to e.g. the MeSH ontology, since abbreviations and alternative spellings of diseases are included in this set.

	Any	*Altered Expression*	*Genetic Variation*	*Regulatory Modification*	*Unrelated*	*Total*
Corpus	1.396	1.750	1.695	186	369	5.396

Table1: Distribution of type of relations in the gene-disease corpus

Inter-annotator agreement was estimated in a similar way like [1] did at the BioCreAtIvE I evaluation conference. We took a small sample of the corpus (5%) and compute the fraction of agreements vs.

disagreements. We marked an association as disagreement if one of the two annotators disagreed [1]. The inter-annotator agreement was estimated to be about 84%.

Labeling

The annotation unit was at the sentence-level, thus, if a GeneRIF was composed of several sentences, these sentences were split automatically and annotated independently from each other. In a further preprocessing step these sentences were tokenized and each token was required to get labeled. The labels were a combination of the entity type (i.e. a disease) plus the relation type holding between the gene/protein (key entity) and the disease. In general, GeneRIF phrases are based on concise phrases, created by domain experts. Another feature of GeneRIF phrases is that the investigated text phrase refers to a key entity, a certain Entrez gene/protein. As a consequence, the gene/protein is already given and all entities mentioned in the phrase encode a relation to the key entity. Thus, the key entity does not have to be labeled.

A toy example with key entity MMP-12 makes this clearer:

> <u>Example E1:</u> MMP-12 polymorphisms are strongly linked with <disease type=”genetic variation”>Alzheimer disease </disease> , but not with <disease type=”unrelated”>breast cancer</disease>.

Note that we use SGML format for labeling, even though the examples here are shown in the MUC (Message Understanding Conference) format. All tokens not inside the *disease* tags are marked as outside (e.g. “MMP-12/O polymorphisms/O” in SGML). The tokens inside an entity are marked with the type of entity plus the relation holding between the entities. In addition, a flag is set, whether we are at the beginning or inside an entity (e.g. Alzheimer/B-gen_var_disease disease/I-gen_var_disease).

Annotation

In this section we describe the rules, which guided the two annotators through the manual labelling process. The annotation scheme for the associations was defined as follows:

***<u>Any</u>*:**
A pair of disease and gene/protein entity is marked as related, if the sentence states clearly a role of the gene in the disease, but there's no further specification, if a certain molecular state of the gene plays a role. Thus, the annotators use this association, if there is clearly a certain role for a gene in a disease, but there's no explicit description that a specific observed state is linked with the disease.

> <u>Example E2:</u> ENG and ALK-1 genes may have roles in <disease type=”any”> hereditary haemorrhagic telangiectasia </disease> in the italian population.

<u>Altered expression:</u>
A pair is marked with this association, if an unusual or altered expression level of the gene/protein was observed for a certain disease. This means that the unusual expression level is somehow linked with a disease. Even though this fact could only be a by-product of the disease, the information could be, for instance, quite valuable for new biomarker candidate identification. Or it could be very likely that at least genes from the same pathway are likely linked etiologically with the disease.

<u>Example E3:</u> Low expression of BRCA1 was associated with <disease type=”altered_exp”> colorectal cancer </disease>.

Assume now that a second gene (different from the key entity BRCA1 in E3) occurs in the sentence and the altered expression statement refers to that gene, while for the key entity there's no such statement. In this case we could only link an *any* association for the key entity or an *unrelated* association if there would be a negative statement.

<u>Genetic variation:</u>
This association indicates that different appearances of the gene/protein are linked with a disease. This could be e.g. polymorphisms, point mutations (such as deletions and substitutions), amino acid substitutions or a SNP. Again, if a genetic variation statement is made for another gene than the key entity, no such relation can be assumed for the key entity unless explicitly stated.

<u>Example E4:</u> The frequency of the <disease type=”gen_var”>hereditary ovarian carcinoma</disease> is attributed to BRCA1 gene mutation.

<u>Regulatory modification</u>
Usually, regulatory modification refers to a gene which is phosphorylated or methylated and thus regulated by such an event in a disease. In the shown example, E-cadherin is the key entity and thus such a modification relation can be extracted.

<u>Example E5:</u> distinct methylation pattern in <disease type=”reg_mod”>bladder cancer</disease> with frequent methylation of RARbeta, DAPK, E-cadherin, and p16.

<u>Unrelated</u>
This association is assigned, if the sentence gives evidence for an independence between a certain state of the key entity gene/protein and a disease. In the example below, E-cadherin is the key entity and thus we can assume an unrelated state association. Of course, this does not automatically mean that these entities are in general not linked, since other publications could come to other conclusion.

<u>Example E6:</u> ETA-2 polymorphisms but not E-cadherin polymorphisms are linked with <disease type=”unrelated_state”>Down Syndrome</disease>.

In what follows, we list the issues the human annotators had to keep in mind additionally, when labeling the data set:

1. Which tokens shall be tagged as part of a disease entity?
As one can see from example E2, the tree tokens *hereditary haemorrhagic telangiectasia* were labelled as one disease entity although in the MeSH ontology only the entry for *Telangiectasis* can be found. We decided to label all additional information as part of the disease entity, if the tokens specify the disease more precisely. Note that this tagging of extra-information can cause problems when named entities are mapped later on to entries of taxonomies/ontologies like e.g. MeSH. However, we believe that these additional disease descriptions represent valuable extra-information.

2. What influence do several gene mentions in a sentence have, when assigning relations between the key entity gene and an occurring disease?
Recall that the task is to infer the relation holding between the key entity gene given by the GeneRIF and a disease. As noted earlier, if several gene mentions of different genes occur in the sentence,

assertions for a more fine grained relation (e.g. a*ltered expression*) for the key entity can only be made if there is an explicit statement about the key entity with respect to the disease. If there are only specific molecular statements about observed states for other genes than the key entity, but not for the key entity itself, the entity pair can only be assigned an *any* or an *unrelated* relation for that sentence (see example E6).

3. How to handle ambiguities of relations?
If it was not clear for an entity pair which relation should be assigned because of ambiguity, then we looked at the PubMed abstract of that GeneRIF. If there was a clear statement, which category is holding for the relation pair, then this association was chosen. If indeed one GeneRIF phrase was stating two possible associations for a disease and a gene (e.g. a methylation event and as a consequence an altered expression event), then we decided to use the association which comes first from a biological point of view (i.e. the association/event which happens in the biological machinery before the other).

4. How to handle nested entities?
Our strategy to handle nested entities was two fold:
Disease mentions in gene/protein names like *breast cancer 2 gene* were not tagged, since nothing is stated explicitly between breast cancer and the key entity gene/protein, whereas disease mentions in cell type names like *prostate cancer cells* were labeled.

Data set creation

We first trained a CRF for disease recognition. The model is based on a separate data set provided by [3]. The 453 genes used in our data set were derived in the following manner:
Genes were randomly drawn exclusively from *Homo Sapiens* from *Entrez Gene* [2] and the attached GeneRIF sentences were added to the text corpus if at least 15% of GeneRIFs for that gene contained one or more disease mentions (recognized by the above mentioned disease CRF). After the number of GeneRIFs had a moderately large size, we skipped this procedure (resulting in 5.720 sentences).
In a next step, two human annotators manually labeled 1000 sentences (each 500) with the previous mentioned predefined types of relations. This fully labeled sub-corpus was used to train a first relational cascaded CRF which was then used to weakly annotate the remaining 4.720 sentences. This weakly labeled text corpus was again fully relabeled by the two human annotators according to the annotation guidelines. The relabeling revealed that a lot of disease mentions and thus relations had been missed (due to a small number of initial training samples) . After the relabeling, a lot of additional new disease mentions came along with and therefore we believe that our disease mentions in the data are quite diverse and general. Mentioned diseases relate to prominent diseases as for example breast cancer, Parkinson or Alzheimer but also cover very specific types such as Adamantiades-Behcet's disease.

References

[1] Colosimo M, Morgan A, Yeh A, Colombe J and Lynette Hirschmann: **Data preparation and interannotator agreement: BioCreAtIvE Task 1B.** In *BMC Bioinformatics* 2005, **6**(Suppl 1):S12

[2] Maglott, D. and Ostell, J. and Pruitt, K. D. and Tatusova, T. : **Entrez Gene: gene-centered information at NCBI.** In *Nucleic Acids Res*, 2005

[3] Rosario B, Hearst M: **Classifying Semantic Relations in Bioscience Texts.** In *Proceedings of the Annual Meeting of Association of Computational Linguistics (ACL '04)* , 2004
